# Supplementary material for: Effect of Fermentation with Streptococcus thermophilus Strains on In Vitro Gastro-Intestinal Digestion of Whey Protein Concentrates
Source: Microorganisms. 2023 Jul 3;11(7):1742. doi: 10.3390/microorganisms11071742 (PMC10386367; doi:10.3390/microorganisms11071742)
Supplement: Supplementary file 1 [file microorganisms-11-01742-s001.zip › microorganisms-2449809-SI.pdf]

Supplementary Table 1: Characteristics of subjects surveyed.

| | 2011 survey n=6528 | 2021 survey n=6534 |
|---|---|---|
| Sex, male, n (%) | 3060 (46.9) | 1059 (53.6) |
| Age (avg ± SD) | 59.8 (13.1) | 61.5 (13.1) |
| Smoking history, n (%) | | |
| Never smoked | 3441 (52.7) | 3554 (54.4) |
| Former smoker | 1819 (27.9) | 2018 (30.9) |
| Current smoker | 1268 (19.4) | 958 (14.7) |
| Attempted to quit smoking, n (%) | 798 (62.9) | 609 (63.6) |
| Perceived level of health, (avg ± SD) ¶ | 7.1 (1.7) | 7.4 (1.7) |
| Report respiratory symptoms, n (%) | 1877 (28.8) | 1618 (24.8) |

Data expressed as mean (standard deviation) or in absolute (relative) frequencies according to the nature of the variable.

¶ Perceived health was evaluated on a scale from 0 to 10 points, with a higher score meaning better perceived health.
